# Principle Superiority and Clinical Extensibility of 2D and 3D Charged Nanoprobe Detection Platform Based on Electrophysiological Characteristics of Circulating Tumor Cells

**DOI:** 10.3390/cells12020305

**Published:** 2023-01-13

**Authors:** Jingyao Chen, Dan Li, Chenqi Zhou, Yuqian Zhu, Chenyu Lin, Liting Guo, Wenjun Le, Zhengrong Gu, Bingdi Chen

**Affiliations:** 1 Institute for Regenerative Medicine, Shanghai East Hospital, The Institute for Biomedical Engineering & Nano Science, Tongji University School of Medicine, Shanghai 200092, China; 2 Fujian Zhihui Medical Technology Co., Ltd., No. 3001, South Shiji Road, Luoshan Street, Quanzhou 362000, China; 3 Shanghai Key Laboratory of Maternal Fetal Medicine, Shanghai Institute of Maternal-Fetal Medicine and Gynecologic Oncology, Shanghai First Maternity and Infant Hospital, School of Medicine, Tongji University, Shanghai 200092, China

**Keywords:** cell electrophysiology, tumor biomarker, electrostatic attraction, nanoprobe dimension, circulating tumor cells

## Abstract

The electrical characteristic of cancer cells is neglected among tumor biomarkers. The development of nanoprobes with opposing charges for monitoring the unique electrophysiological characteristics of cancer cells. Micro-nano size adsorption binding necessitates consideration of the nanoprobe’s specific surface area. On the basis of the electrophysiological characteristics of circulating tumor cells (CTCs), clinical application and performance assessment are determined. To demonstrate that cancer cells have a unique pattern of electrophysiological patterns compared to normal cells, fluorescent nanoprobes with opposing charges were developed and fabricated. Graphene oxide (GO) was used to transform three-dimensional (3D) nanoprobes into two-dimensional (2D) nanoprobes. Compare 2D and 3D electrophysiological magnetic nanoprobes (MNP) in clinical samples and evaluate the adaptability and development of CTCs detection based on cell electrophysiology. Positively charged nanoprobes rapidly bind to negatively charged cancer cells based on electrostatic interactions. Compared to MNPs(+) without GO, the GO/MNPs(+) nanoprobe is more efficient and uses less material to trap cancer cells. CTCs can be distinguished from normal cells that are fully unaffected by nanoprobes by microscopic cytomorphological inspection, enabling the tracking of the number and pathological abnormalities of CTCs in the same patient at various chemotherapy phases to determine the efficacy of treatment. The platform for recognizing CTCs on the basis of electrophysiological characteristics compensates for the absence of epithelial biomarker capture and size difference capture in clinical performance. Under the influence of electrostatic attraction, the binding surface area continues to influence the targeting of cancer cells by nanoprobes. The specific recognition and detection of nanoprobes based on cell electrophysiological patterns has enormous potential in the clinical diagnosis and therapeutic monitoring of cancer.

## 1. Introduction

As a crucial component of liquid biopsies, the detection of circulating tumor cells (CTCs) continues to face numerous obstacles [1,2]. The most difficult aspect of CTC detection is isolating the exceedingly rare CTCs from many blood cells [3,4]. The optimal enrichment strategy selectively differentiates cancer cells from normal cells and applies it to all cancer types [5]. Currently, immunoaffinity based on cell surface antigens [6] and separation methods based on physical factors such as cancer cell size [7], density, and charge-to-mass ratio [8] are the current standard methods for detecting CTCs. The most significant issue with these CTCs capture approaches is that the features of cancer cells used for CTC detection are not unique to cancer cells and are not generally applicable to other cancer types, resulting in a poor broad spectrum and low sensitivity [9,10].

The electrophysiological pattern of cancer cells is notably distinct from those of normal cells [11]. The capacity of cancer cells to generate energy via the glycolysis pathway is a characteristic that distinguishes them from normal cells [12]. The “Warburg effect” causes cancer cell surfaces to be negatively charged [13,14]. As a distinct electrophysiological mode of cancer cells, it represents a promising solution to the problem of CTC capture across a broad spectrum. However, the clinical viability of this concept has not yet been evaluated.

As a result of electrostatic contact, binding is extremely rapid [15]. Boosting the nanoprobes’ binding probability to cancer cells by increasing their specific surface area has been studied. Numerous oxygen-containing functional groups are present in graphene oxide (GO), endowing it with exceptional properties including high dispersibility and compatibility with polymers [16]. Futhermore, oxygen-containing functional groups on the GO surface and polymer matrix can increase the interfacial contact force via hydrogen bonding [17]. In this study, an interface for the conversion of 3D nanoprobes to 2D was designed utilizing GO’s distinct nanostructure, large surface area, solubility in aqueous solution, and ease of modification of oxygen-containing functional groups [18]. Few comparative interface contact area studies have been undertaken in prior reports (mainly for biosensing applications [19]). Consequently, considerably more work is necessary.

Rapid advances in nanotechnology have been made in the early diagnosis and treatment of cancer [20]. Combining nanotechnology and biomedicine, nanomedicine demonstrates superior properties in cancer diagnosis and treatment [21]. The design and synthesis of nanoprobes for the enrichment and isolation of CTCs focuses primarily on the following three properties: first, a high specific surface area, which is advantageous for increasing the likelihood of contact between nanoprobes and cells and, second, superparamagnetism and fluorescence for CTC monitoring and enrichment separation; third, a low nanoprobe dosage and minimal toxicity, which facilitates the subsequent application of CTCs. Lastly, the advancement of nanoprobes and detection principles based on the electrophysiological feature detection platform for circulating tumor cells was studied.

## 2. Results

### 2.1. Unique Electrophysiological Pattern of Cancer Cells and Design of Electrophysiological Nanoprobes for Monitoring

**Figure 1:**
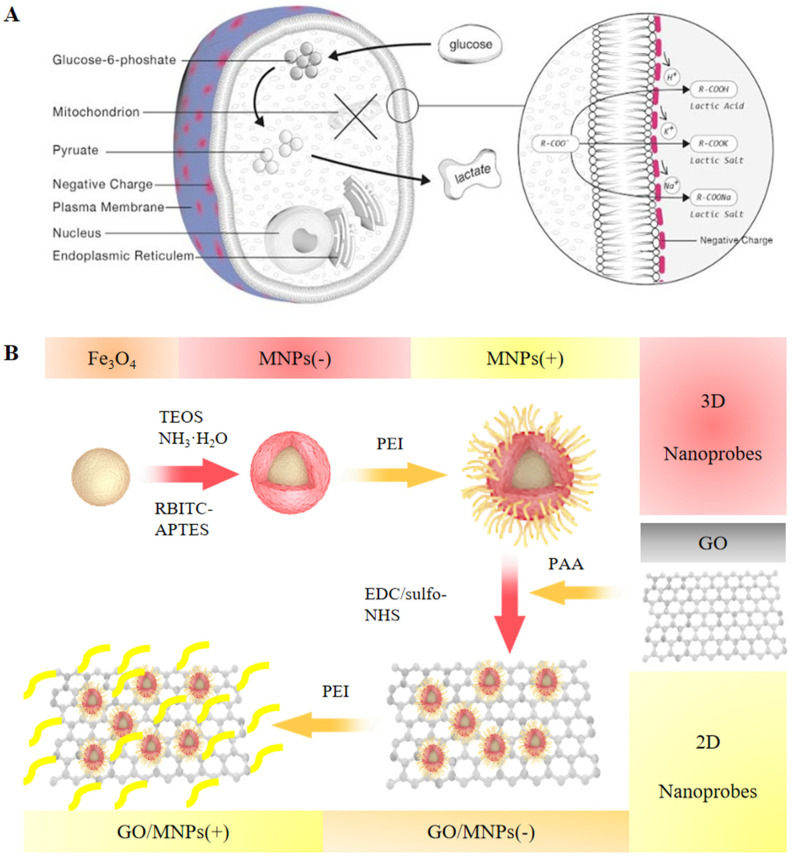
Design of three-dimensional (3D) and two-dimensional (2D) nanoprobes for cellular electrophysiological property detection. (**A**) Due to differences in glucose metabolic pathways, normal cells and cancer cells exhibit distinctive electrophysiological patterns. (**B**) The synthetic design of 2D and 3D nanoprobes are represented schematically. Nanoprobes with 2D are GO/MNP(+) and GO/MNP(-). Nanoprobes with 3D include MNP(+) and MNP(-). “+” indicates the nanoprobe has a positive charge, while “-” indicates a negative charge.

Without employing any molecular biomarkers, a set of electrostatically charged, fluorescent, and superparamagnetic nanoprobes was designed to specific targeting of cancer cells. Figure 1A shows that the activities of glucose metabolism have a significant influence on the surface charge of cancer cells. Lactate production increased as a result of enhanced glycolysis in cancer cells. It is known that the produced lactate anions remove positive ions from the cell surface, leaving negative changes [13]. This unusual metabolic function is responsible for the perpetuation of negative surface charges on cancer cells. By synthesis of nanoprobes with distinct charge patterns (Figure 1B), the electrophysiological properties of cells can be measured. Simultaneously, 2D and 3D nanoprobes are developed to investigate the effect of spatial bonding surfaces on electrostatic attraction. Using PEI, silica-coated Fe_3_O_4_ magnetic nanoparticles with negatively charged were converted into positively charged MNPs(+). PAA was used to modify the surface of GO, increasing its carboxyl content and dispersibility. The brown spheres represent the core of Fe_3_O_4_ The color red represents the silica coating and the rhodamine B alteration. The yellow tail is PEI modified with amino acids. The gray sheet is GO. The above elements are the constituent components of GO/MNPs. PEI modification was performed to obtain GO/MNPs(+).

### 2.2. Characterization of Electrophysiological Nanoprobes

**Figure 2:**
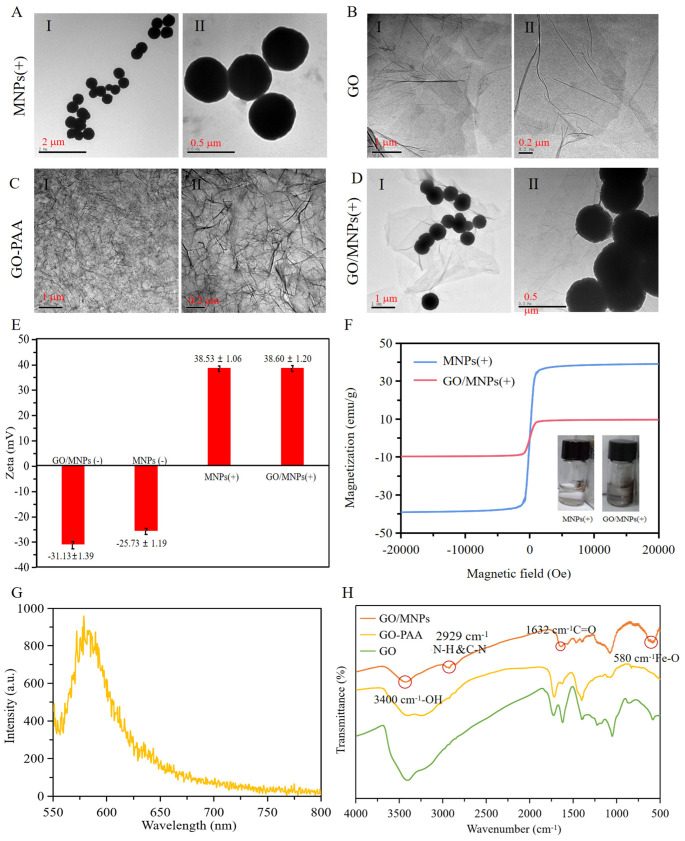
Characterization of electrophysiological nanoprobes. TEM images of (**A**) MNPs(+); (**B**) GO; (**C**) GO-PAA; (**D**) GO/MNPs(+); (**E**) Zeta potential of nanoprobes in an aqueous solution; (**F**) magnetic properties and hysteresis loops of MNPs(+) and GO/MNPs(+); Magnetization curve of both MNPs(+) and GO/MNPs(+) obtained through a VSM; The curve shows the superparamagnetism of MNPs(+) and GO/MNPs(+); (**G**) fluorescence spectra of GO/MNPs; (**H**) infrared spectra of GO, GO-PAA and GO/MNPs.

As shown in Figure 2A and Supplementary Material Figure S1, the average hydrated particle size of MNPs(+) is 756.16 ± 75.96 nm, and its microscopic morphology is spherical. TEM images of GO (Figure 2B) and GO-PAA (Figure 2C) reveal a layer structure with a wrinkled appearance. These bends and folds contribute to the stability of its two-dimensional structures from the perspective of macrodynamics; from the perspective of microstructure, these folds can further form pores and fold channels, thereby increasing the specific surface area of graphene oxide, which can be used as an adsorption material. In Figure 2D, the spherical MNPs(+) nanoparticles are bonded to the GO-PAA of the two-dimensional flake layer to form GO/MNPs(+). As shown in Figure 2E, the zeta potential of GO/MNPs(-) is −31.13 ± 1.39 mV, and the zeta potential of MNPs(-) is −25.73 ± 1.19 mV. The zeta potential of GO/MNPs(+) is +38.60 ± 1.20 mV. Due to the electrostatic effect, the PEI-modified nanoprobes of two different dimensions have positive surface charges and, in theory, bind to cancer cells with negative surface charges. Figure 2F depicts the hysteresis loops of MNPs(+) and GO/MNPs(+); both MNPs(+) and GO/MNPs(+) are superparamagnetic. GO/MNPs(+) has a lower saturation magnetization than MNPs(+), but after the capture of cancer cells, its magnetism is sufficient for magnetic separation. The emission wavelength of rhodamine B is 580 nm. GO/MNPs(+) fluorescence peaks at 580 nm (Figure 2G), indicating that rhodamine B is encapsulated within GO/MNPs(+). The stretching vibration peak of -OH is observed at 3400 cm^−1^ for GO, GO-PAA, and GO/MNPs(+), whereas the stretching vibration of the C=O bond is observed near 1632 cm^−1^. The peaks on GO are associated with −OH and −COOH. GO/MNPs(+) exhibits N-H and C-H stretching vibration peaks at 2929 cm^−1^ and Fe-O stretching vibration peaks at 580 cm^−1^. Figure 2H demonstrates that MNPs(+) was successfully grafted to GO-PAA, resulting in the formation of GO/MNPs(+).

### 2.3. Specific Targeting of Cancer Cells Based on the Cellular Electrophysiological Characteristics

**Figure 3:**
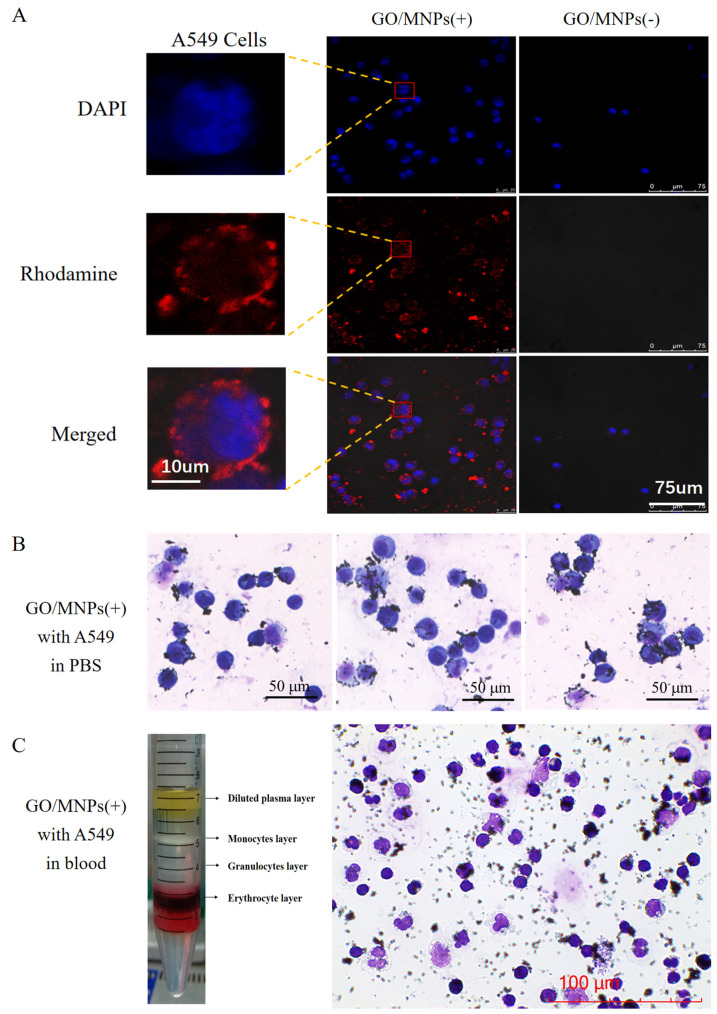
Monitoring the electrophysiological characteristic of cancer cells using charged nanoprobes. (**A**) A549 cells binding status to GO/MNPs(+) and GO/MNPs(-). The microscopic observation of the specific targeting of GO/MNPs(+) to A549 cells in (**B**) PBS or (**C**) simulated blood samples (cancer cells spiked into healthy blood samples), and the digital picture of different cell types in blood samples can be separated by gradient centrifugation for pretreatment.

To examine the electrophysiological properties of the cellular surface, nanoprobes have been developed. Observation of the binding of GO/MNPs with variously charged properties to cells provided additional evidence that the surface charges of cancer cells. Figure 3A shows the binding of GO/MNPs(+) and GO/MNPs(-) to A549 cells as visualized by confocal laser scanning microscopy. The nucleus of A549 cells had blue fluorescence following DAPI labeling, while the GO/MNPs(+) and GO/MNPs(-) composite nanoprobes displayed red fluorescence. GO/MNPs(-) and A549 cells after co-incubation and washing with PBS, the amount of red fluorescence was very low, indicating little binding between GO/MNPs(-) and cancer cells; however, GO/MNPs(+) and A549 cells after co-incubation and washing with PBS, the surface of A549 cells still displayed strong red fluorescence, indicating a proximity effect. Electrostatic adsorption was demonstrated to be the mechanism for the binding of GO/MNPs(+) to A549 cells. The surface electrostatic pattern of metabolically active cancer cells can be exploited to recover cancer cells from circulating blood and other solutions. In order to simulate blood samples from cancer patients, cancer cells were spiked to healthy human blood samples in addition to having cancer cells in PBS as a control. Blood samples containing reference cancer cells are gradient separated to remove red blood cells preferentially, followed by enrichment with nanoprobes for specific targeting. Targeting binding of nanoprobes to A549 cells was observed under the microscope in PBS and simulated blood (Figure 3B,C). Similar target binding behavior was observed with MDA-MB-231-GFP (Supplementary Material Figure S2).

### 2.4. Analysis of the Effect of 2D and 3D Nanoprobe Spatial Specific-Surface-Area on Electrostatic Attraction Binding

**Figure 4:**
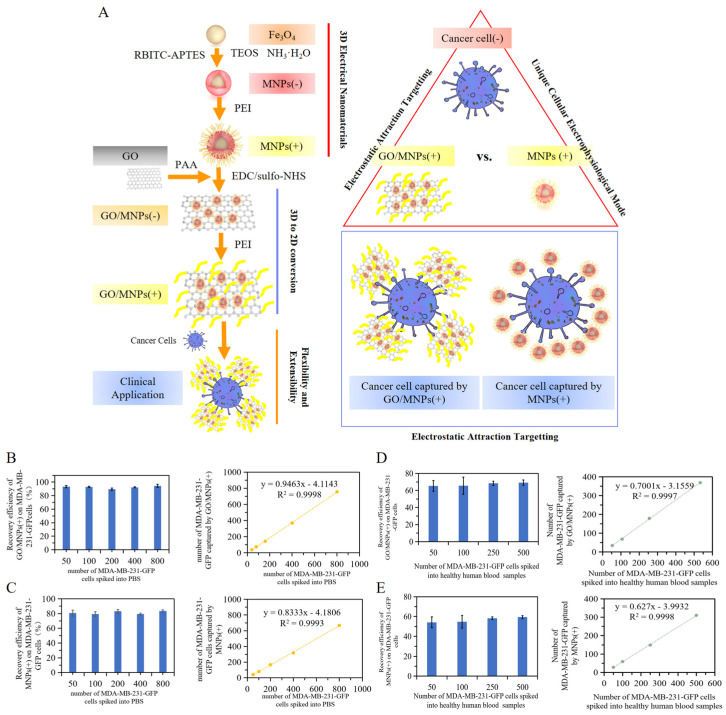
Comparation of 2D and 3D nanoprobe spatial specific-surface-area on electrostatic attraction binding. (**A**) conceptual diagram of interactions between GO/MNPs(+) and MNPs(+) with cancer cells. Recovery rates of (**B**) GO/MNPs(+) and (**C**) MNPs(+) on different numbers of gradient MDA-MB-231-GFP cancer cells in PBS; recovery rates of (**D**) GO/MNPs(+) and (**E**) MNPs(+) with different numbers of gradient cancer cells added to peripheral blood samples of healthy people. Linear correlation analysis between the number of GO/MNPs(+) and MNPs(+) recovered MDA-MB-231-GFP cancer cells and the number of MDA-MB-231-GFP cancer cells added to internal reference.

A predetermined number of internal reference cells can be utilized to assess the performance of 2D and 3D nanoprobes in capturing tumor cells. There was no statistical difference between GO/MNPs(+) and MNPs(+) on the comparison of recovery rates between each experimental sample with different number gradients (*t*-test, *p* > 0.05); The mean recovery rate of GO/MNPs(+) for MDA-MB-231-GFP cells in PBS was 92.52 ± 1.64%, which was significantly higher than the mean recovery rate of MNPs(+) for MDA-MB-231-GFP cancer cells in PBS, which was 81.19 ± 1.76%. GO/MNPs(+) was found to be more effective than MNPs(+) at capturing MDA- MB-231-GFP cancer cells in PBS (Figure 4B,C). The capture efficiency of GO/MNPs(+) and MNPs(+) upon the addition of MDA-MB-231-GFP cancer cells with a number of cancer cells up to 500 (50, 100, 250, 500) as internal reference cancer cells in the peripheral blood of healthy subjects (Figure 4D,E). The mean recovery rate of GO/MNPs(+) for different number gradients of MDA-MB-231-GFP cancer cells in blood samples from simulated patients was 67.15 ± 1.67% compared with 56.65 ± 1.57% for MNPs(+). The number of recovered cells of GO/MNPs(+) and MNPs(+) for MDA-MB-231-GFP cancer cells increased with the addition of MDA-MB-231-GFP cancer cells; however, based on the slope of their linear correlation equations, the sensitivity of GO/MNPs(+) to capture cancer cells in simulated patient blood samples was greater than that of MNPs(+), with a slope of 0.7001. Results indicated that GO/MNPs(+) was more effective than MNPs(+) at capturing MDA-MB-231-GFP cancer cells in both PBS and simulated blood samples.

### 2.5. Clinical Flexibility Based on Electrophysiological Pattern of Circulating Tumor Cells Detection

**Figure 5:**
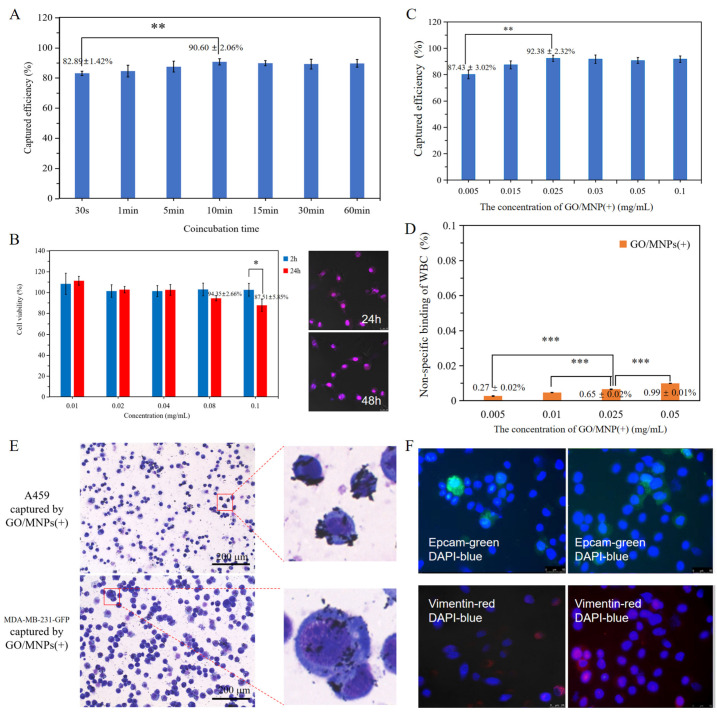
Principle scalability of the charged nanoprobe detection platform based on tumor cell electrophysiological characteristics. (**A**) detection speed, the capture efficiency of GO/MNPs(+) on A549 cells under different co-incubation time conditions (SD, *n* = 3, *t*-test: *p* < 0.01 **); (**B**) biosafety and reculture, cell viability of A549 cells co-cultured with GO/MNPs(+) at different concentrations for 2 h and 24 h (SD, *n* = 5, *t*-test: *p* < 0.05 *), and captured cells activity at 24 h and 48 h; (**C**) nanoprobe usage and capture efficency, capture efficiency of A549 cells at different concentrations of GO/MNPs(+) (SD, *n* = 3, *t*-test: *p* < 0.01 **); (**D**) detection specificity, nonspecific targeting efficiency of the combination of nanoprobes and white blood cells (WBC) (SD, *n* = 3, *t*-test: *p* < 0.001 ***); (**E**) The cancer cells with different physical sizes were captured by GO/MNPs(+) (bar = 200 um); (**F**) identification by immunofluorescence of epithelial and mesenchymal cancer cells captured by GO/MNPs(+).

The current detection principles of CTCs are mostly based on the principle of immune affinity [22,23] or physical size filter [24,25]. Exploration of the clinical utility of CTCs detection based on the unique electrophysiological pattern of cancer cells is warranted. Figure 5A illustrates the electrostatic bonding efficiency of GO/MNPs(+) to A549 cells under different co-incubation conditions. At 30 s of co-incubation between nanoprobe and cells, the GO/MNPs(+) captures efficiency for A549 cells electrostatic binding was 82.89 ± 1.42%. As the duration of co-incubation increased, the capture efficiency of GO/MNPs(+) on A549 cells increased. At 10 min, the GO/MNPs(+) capture rate on A549 cells was 90.60 ± 2.06%. The capture rate did not continue to increase as the co-incubation time was increased, indicating that 10 min was the optimal co-incubation time for maximum capture efficiency. In subsequent experiments, the co-incubation time was therefore set to 10 min. The cell viability after co-incubation of various concentrations of GO/MNPs(+) with A549 cells for 2 h and 24 h is shown in Figure 5B, whereas the captured cancer cells still exhibited viability after 48 h. With concentrations below 0.1 mg/mL and co-incubation times below 2 h, nanoprobes exhibited negligible toxicity to cancer cells which is advantageous for clinical applicability. Figure 5C shows the capture efficiency of various GO/MNPs(+) concentrations on A549 cells. When the concentration of GO/MNPs(+) nanoprobe was increased to 0.030 and 0.05 mg/mL, the capture rate of A549 cells remained relatively stable. In all subsequent experiments, the concentration of the GO/MNPs(+) nanoprobe was set to 0.025 mg/mL in consideration of the principle of the minimum amount of material and the background clarity of the post-production slides. The leukocyte adsorption rate on GO/MNPs(+) nanoprobes with varying concentrations in Figure 5D. When the GO/MNPs(+) concentration is 0.005 mg/mL, the leukocyte adsorption rate is 0.27 ± 0.02%. The adsorption rate continued to increase as the concentration of GO/MNPs(+) increased. The adsorption rate on leukocytes increased to 0.99 ± 0.01% as the GO/MNPs(+) concentration continued to rise to 0.05 mg/mL. Monitoring based on the electrophysiological pattern of circulating tumor cells can simultaneously capture cells of different physical sizes, as well as epithelial and mesenchymal cancer cells (Figure 5E,F). A broad-spectrum analysis utilizing the cell electrophysiological signature capture platform revealed that GO/MNPs(+) and MNPs(+) had targeting effects on five distinct types of cancer cells (Supplementary Material Figure S3).

### 2.6. Clinical Validation of Electrophysiological Nanoprobes

**Figure 6:**
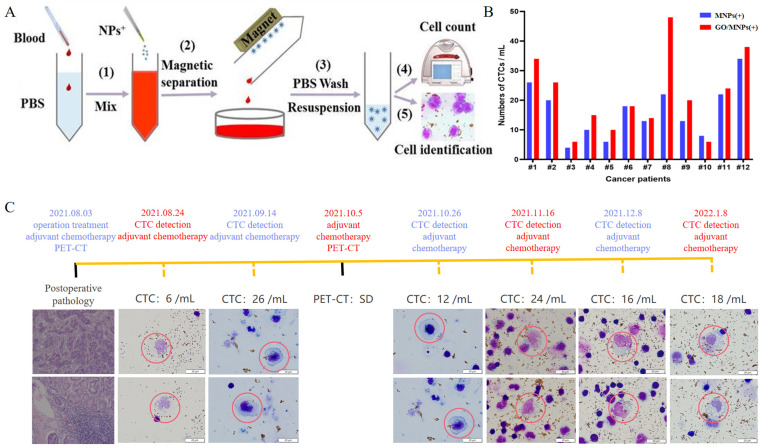
Validation of charged nanoprobes for the detection of circulating tumor cells in the clinical. (**A**) illustration conceptual of electrostatic nanoprobes for detecting CTCs; (**B**) CTC detection results of MNP(+) and GO/MNP(+) nanoprobes in clinical samples; (**C**) detection results from the same patient at different time points. Pathological morphological analysis of cells was performed under the microscope (bar = 20 um).

Figure 6A shows the flowchart of GO/MNP(+) for CTC detection. Mix by gently inverting 2 mL of peripheral blood with 2 mL of 1× PBS, and slowly add 4 mL of the diluted blood sample to the separation solution. After gradient centrifugation, the granulocytes and monocytes layers were aspirated (Figure 3C, left digital picture). To prevent endocytosis of nanomaterials by cells, electrophysiological nanoprobes were incubated with cells for 10 min at 4 °C. Magnetic separation was performed to resuspend CTCs. Finally, use Diff-Rapid staining to determine the morphology of the cells. After drying the slides, photographs were taken under a microscope. Figure 6B shows the results of CTC detection using MNPs(+) and GO/MNPs(+) for each patient. Clinical information is shown in Supplementary Material Table S1. Electrophysiological nanoprobes successfully captured CTCs from patients with multiple types of cancer (*n* = 12). As shown in Figure 6C, the electrophysiological nanoprobe was used to monitor the number and morphological changes of CTCs at various stages of the same patient. After each course of chemotherapy, the number of CTCs fluctuated in a stable range (6–26/mL) without a sustained upward trend. In the early stage of adjuvant chemotherapy, the diameter of CTCs was >15 μm, mostly oval, with hyperchromatic nuclear atypia and thickened nuclear membrane. With the increase of chemotherapy times, the coloring of the nuclei of CTCs became lighter, and the cytoplasm had vacuoles. The residual target binding of nanoprobes to cells has a negligible effect on diagnostic outcomes. The purity of CTCs in total captured cells in clinical samples is shown in Supplementary Figure S4.

## 3. Materials and Methods

### 3.1. Synthesis of 2D and 3D Nanoprobe Transition Interface

GO and polyacrylic acid (PAA) were purchased from wins Czech Optoelectronics and McLean Technology Co., Ltd. (Prague, Czech Republic). Surface modification of GO using the literature as a guide [26]. The following is the synthesis: 20 mg of GO is weighed in a mortar, the powder is triturated for 10 min, 20 mL of deionized water is added, and the mixture is ultrasonically dispersed for 40 min to yield 1 mg of GO per milliliter of water. In addition, 200 mg PAA (MW = 1800), 20 mL of deionized water, and 10 min of ultrasonication until complete dissolution yield a PAA solution of 10 mg/mL. The dissolved PAA was added dropwise to an equal volume of GO ultrasonic dispersion in an aqueous dispersion, and the ultrasonic dispersion continued for 40 min with the temperature not exceeding 28 °C. After ultrasound, 17,000× *g*, and 20 min of centrifugation, the supernatant was discarded and the process was repeated three times to eliminate unreacted PAA. Freeze-dried graphene oxide-polyacrylic acid(GO-PAA) for usage.

### 3.2. Synthesis of Nanoprobes with Opposing Electrical Modes

Add 10 mg of GO-PAA to 10 mL of pH 5.5 to 6.0 MES buffer and sonicate for 20 min. Add 1 mL of a 10 mg/mL aqueous solution of EDC to 1 mL of a 10 mg/mL aqueous solution of GO-PAA, then add 1 mL of a 10 mg/mL aqueous solution of sulfo-NHS to the above reaction system for 15 min at room temperature. To remove the MES buffer, centrifuge for 20 min at 17,000× *g*. The precipitate was resuspended in phosphate buffered saline (pH = 7.4), and 3 mL of a 1 mg/mL MNPs(+) aqueous solution was added. After 12 h of shaking reaction, the magnets were separated, and deionized water was utilized. GO/MNPs(-) were produced after three washes. Until usage, freeze-dried and stored at 4 °C. Surface modification of negatively charged GO/MNPs with PEI to generate positively charged GO/MNPs, or GO/MNPs(+). Add 20 mL of 1 mg/mL GO/MNPs(-) to 20 mL of PEI aqueous solution(10 mg/mL) and continue sonicating for 20 min. The resulting homogeneous suspension was placed on a constant-temperature shaker at room temperature and kept dark for 12 h. Magnets were separated and rinsed three times with deionized water to produce PEI-modified graphene oxide/magnetic beads, also known as GO/MNPs(+). Until usage, freeze-dry and store at 4 °C.

### 3.3. Characterization of the 2D and 3D Electrophysiological Nanoprobes

Weigh a sufficient amount of GO, GO-PAA, MNPs(+), and GO/MNPs(+) powders, resuspend them in deionized water, and disperse them uniformly with ultrasound. The micro-morphology of each specimen was examined using a transmission electron microscope (TEM). Select at least 150 nanoparticles at random from the electron microscope images in order to study the particle size distribution and determine the mean particle size. Utilizing the Zeta sizer Nano-ZS90 potential analyzer, the surface potentials of MNPs(-), MNPs(+), GO/MNPs(-), and GO/MNPs(+) nanoparticles were determined. Utilizing a SQUID-VSM magnetic measuring equipment, the saturation magnetization of MNPs(+) and GO/MNPs(+) nanomaterials was determined. Take 1 mL of 0.1 mg/mL GO/MNPs(+) in a transparent cuvette in order to determine the fluorescence properties of nanomaterials. The excitation light has a scanning speed of 1200 nm/min and a wavelength of 540 nm. The UV-visible spectrophotometer model Cary 50 was used to detect the absorption spectra between 200 and 1100 nm. Simultaneously, the UV-visible absorption spectra of PAA, MNPs(+), and GO/MNPs(+) were determined. Using an FTIR Nexus 6700 (Nicolet) Fourier infrared spectrometer, the chemical makeup and functional groups of the materials were analyzed. Take 2 mg of GO, GO-PAA, or GO/MNPs(+), combine it with 200 mg of potassium bromide(KBr), place the mixture in an agate mortar, and grind it clockwise under an infrared light. The instrument specifications are as follows: 4 cm^−1^ resolution, 64 times of scanning, and 4000–400 cm^−1^ scanning range. The iron content of GO/MNPs(+) and MNPs(+) was determined using Optima 2100 DV ICP-OES. Sample pretreatment: 100 uL GO/MNPs(+) aqueous solutions(1 mg/mL) and MNPs(+) aqueous solutions (1 mg/mL) were respectively distributed in 900 uL aqua regia (V concentrated hydrochloric acid:V concentrated nitric acid = 3:1) and digested. Make up to 10 mL of deionized water overnight. Preparation of varying quantities of iron standard solutions: Take 1 mL of the iron standard (1000 g/mL) and dilute it with 1% nitric acid solution to provide iron ion concentrations of 100 ppm, 10 ppm, and 1 ppm, respectively. As a control, the 1% nitric acid solution was employed.

### 3.4. Validation of Nanoprobe Targeting Recovery by Spiked Cells

Cell culture reagents were bought from GIBCO Invitrogen Corporation/Life Technologies Life Sciences. A459 and MDA-MB-231-GFP cells were grown in DMEM and DMEM/F12 media with 10% fetal bovine serum and 1% penicillin-streptomycin solution. Cells were collected and stained with a cell tracking dye (Invitrogen, CellTracker Green CMFDA, C7025). These fluorescence-tracked cells were then utilized for capture efficiency trials. For cell spiking, cells were diluted in serum-free media beginning with a concentration of 1–10^5^ cells/mL. In addition, 1 mL of the concentrated cell solution was transferred to a 96 well plate with low attachment. After counting the transplanted cells under a microscope, they were immediately pipetted into 1 mL of whole blood. After removing the cells from the 96-well plate, the cells at the same location were counted. The total number of cells injected into blood was determined by subtracting the cells left behind from the originating site. A specific number of cells were added to the suspension, and a magnet was utilized to attract the cell-capturing composite. The composite was washed three times with PBS to remove the supernatant, and then the trapped cancer cells were collected. Under a microscope, the abnormal morphology and number of cells collected by the electrophysiological nanoprobe were directly examined, and the capture efficiency was calculated.

### 3.5. Cytotoxicity Detection of Nanoprobes

Six distinct concentration gradients were established, and GO/MNPs(+) were dispersed in DMEM blank medium and prepared for use at concentrations of 0, 0.01, 0.02, 0.04, 0.08, and 0.1 mg/mL. Each well of the 96 well plate contained approximately 8000 cells. After the cells were plastered, 100 uL of the material suspension at various concentrations were added to each well. Incubate for an additional 2 h and 24 h. The Cell Titer-Glo^TM^ assay working solution was produced by dissolving Cell Titer-Glo^TM^ buffer at room temperature and thoroughly mixing Cell Titer-Glo^TM^ powder until the powder was completely dissolved. Mix and shake gently for 2 min to induce cell lysis, incubate for 10 min at room temperature to stabilize the luminescence, and then use a Spectra Max M5 multifunctional enzyme marker to measure the luminescence intensity.

### 3.6. Clinical Blood Specimen Collection

Blood samples were drawn from patients with tumors and healthy donors under an IRB-approved protocol (No. 2020-126). Blood samples are collected from cancer patients prior to surgery, with the vein at the elbow fossa serving as the collection site. Each specimen was collected in EDTA tubes and processed within twenty-four hours. All relative study was approved by the Institutional Review Board of Shanghai Dongfang Hospital. Written informed consent was obtained from all patients.

### 3.7. Detection of CTCs

CTCs were isolated from the peripheral blood using magnetic separation and density gradient centrifugation. The blood is diluted, added to the upper layer of the cell separation solution, and centrifuged to obtain several stratifications, in the order of plasma layer, single nucleated cell layer, neutrophil layer, and red blood cell layer. After collecting the white film layer, MNPs(+) or GO/MNPs(+) were applied to collect CTCs by magnetic separation.

### 3.8. Statistical Analysis

Data analysis was performed using the one-way analysis of variance provided by Excel to analyze the differences between the sample groups. Error line SD, *n* = 3, *t*-test: *p* < 0.05 is a statistical difference, marked *, *p* < 0.01 is a significant statistical difference, marked **, *p* < 0.001 is a significant statistical difference, marked ***.

## 4. Discussion

Due to the heterogeneity of tumors [27,28], the biological traits and mutation gene spectrum of tumor cells in various sections are inconsistent. The high complexity and diversity of malignant tumors in the development process make it difficult to find a uniform tumor biomarker [29,30], which is the main issue in the current research on capturing CTCs [29]. In the past, the emphasis was placed on discovering the specific protein expression and the physical size difference between cells, while research on the electrophysiological characteristics of cancer cells was limited [31].

As a result of the “Warburg effect”, cancer cells generate a large amount of lactate ions in the glycolysis pathway [32], which can combine with cations (H^+^, Na^+^) on the surface of the cell membrane to form lactic acid or sodium lactate during the process of secretion [33]. While lactate removes a large number of cations from the cell membrane, it leaves behind a large amount of anions, resulting in a strong negative charge on the surface of cancer cells [13]. The glycolytic pathway is primarily responsible for the presence of a large number of negative charges on the surface of cancer cells (Figure 1A). WBC were layered and isolated from peripheral blood and then co-incubated with surface-modified positively charged nanoprobes. The results indicate that nanoprobes with positively charged surfaces are nearly incapable of adsorbing WBC (Figure 5D). The glycolytic pathway distinguishes cancer cells from normal cells in their surface electrophysiology. Electrophysiology on the surface of cancer cells exhibits a distinct pattern from that of leukocytes, which is anticipated to serve as a new target for circulating tumor cells’ detection platforms.

Determining the presence and number of circulating tumor cells in peripheral blood can provide crucial prognostic and therapeutic response pattern information. However, due to their inherent rarity and heterogeneity, developing a CTC detection method with clinically significant specificity and sensitivity is extremely difficult. Based on the broad-spectrum characteristics of negative charges on the surface of cancer cells, graphene oxide/magnetic beads’ composite particles (Figure 1B, Supplementary Material Figure S5) were designed and prepared, GO/MNPs, which were coated with Rhodamine SiO_2_ by graphene oxide and surface-modified positive charges for CTC capture. It was demonstrated that the electrophysiological nanoprobe GO/MNPs(+) could capture cancer cells effectively (Figure 4B,D) and trace CTCs in 2 mL of human peripheral blood (Figure 6B). The capture rate of A549 cells was highest at 92.38 ± 2.32% when the nanoprobe concentration was 0.025 mg/mL, and the co-incubation time was 10 min in PBS (Supplementary Material Figure S6 and Figure 5A,C). A trace varying number of CTCs in peripheral blood from clinical cancer patients can also be detected based on cancer progression of the patients (Figure 6C). By designing nanoprobes with charged properties, CTCs from both simulated and clinical blood samples were successfully captured and monitored.

In the context of micro-nanoscale adhesion, the effect of the contact interaction interface is considered. As targeting nanoprobes, GO/MNPs(+) and MNPs(+) were used to compare the capture efficiency of cancer cells in PBS and simulated patient blood samples (Figure 4A). Compared to MNPs(+), GO/MNPs(+) demonstrated a higher capture efficiency for MDA-MB-231-GFP cells with varying gradient numbers. In PBS, the average recovery rate of GO/MNPs(+) was 92.52 ± 1.64%, higher than the average recovery rate of MNPs(+) on cancer cells of 81.19 ± 1.76% (Figure 4B,C). In simulated patient blood samples, the average recovery rate of GO/MNPs(+) to MDA-MB-231-GFP cancer cells was 67.15 ± 1.67%, which was also superior to the average recovery rate of MNPs(+) under the same conditions, which was 56.65 ± 1.5% (Figure 4D,E). The result shows that nanoprobe with a larger specific surface area is more conducive to micro-nanoscale target binding.

A wide variety of CTC subtypes can be obtained based on the principles of cellular electrophysiology. Compared to the antigen–antibody approach, the platform based on the electrophysiological signature of cancer cells as a targeting principle has the benefits of low cost, rapid electrostatic targeting binding procedure for capturing CTCs, and a broad-spectrum of cancer types. Broad-spectrum validation of cancer cells captured by GO/MNPs(+) and MNPs(+) was performed in PBS and clinical samples (Supplementary Material Figure S3 and Table S1).

Furthermore, a circulating tumor cells capture platform utilizing electrophysiological principles can capture cancer cells of different sizes (Figure 5E) and not miss EMT-transformed tumor cells (Figure 5F), which is a preponderance in principle. Even if cancer cells are separated by magnetism, they continue to bind with the electrophysiological nanoprobes, which has no impact on the observation and monitoring of cell pathological morphology (Figure 6C). In the clinical field, genomic analysis is essential for more usability of CTCs and is finally applicated to cancer diagnosis and prognosis [34,35]. GO/MNPs(+) have good biosafety (Figure 5B), which provides more possibilities for downstream CTC analysis. Future CTC capture purification improvements are under consideration for diverse downstream applications. A platform for the electrophysiological detection of circulating tumor cells has the potential to be used in clinical cancer screening to improve the understanding of cancer metastasis and evaluate the prognosis of cancer patients.

## Data Availability

Data is contained within the article or supplementary material.

## Supplementary Materials

The following supporting information can be downloaded at: https://www.mdpi.com/article/10.3390/cells12020305/s1.
