# Supplementary material for: Principle Superiority and Clinical Extensibility of 2D and 3D Charged Nanoprobe Detection Platform Based on Electrophysiological Characteristics of Circulating Tumor Cells
Source: Cells. 2023 Jan 13;12(2):305. doi: 10.3390/cells12020305 (PMC9856308; doi:10.3390/cells12020305)
Supplement: Supplementary file 1 [file cells-12-00305-s001.zip › cells-2061845-SI.pdf]

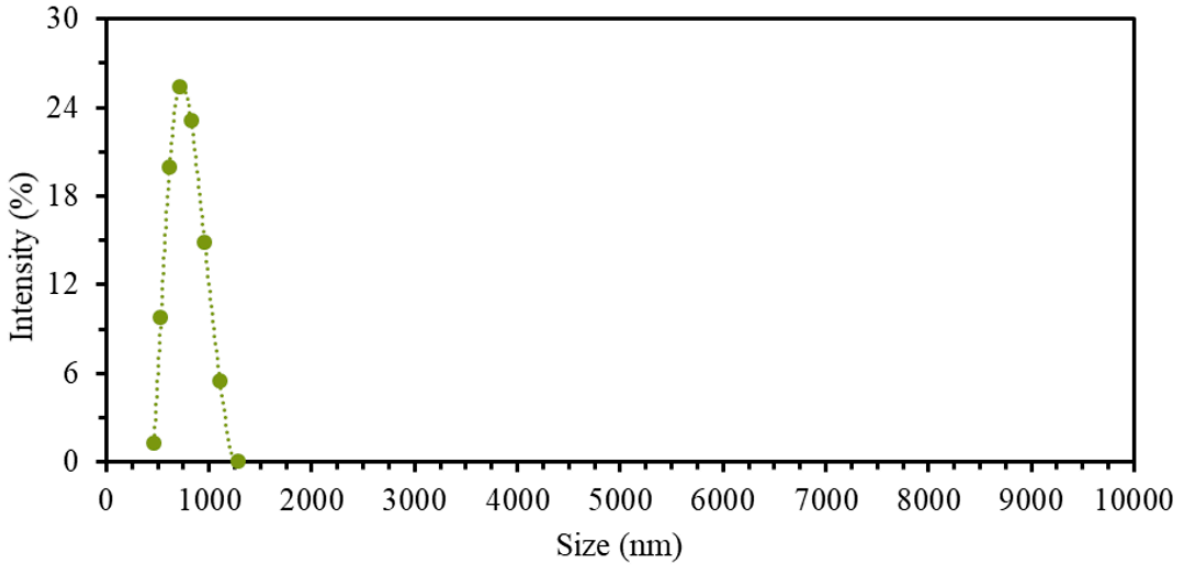

Supplementary Figure S1. The size distribution of MNPs(+) nanoprobe.

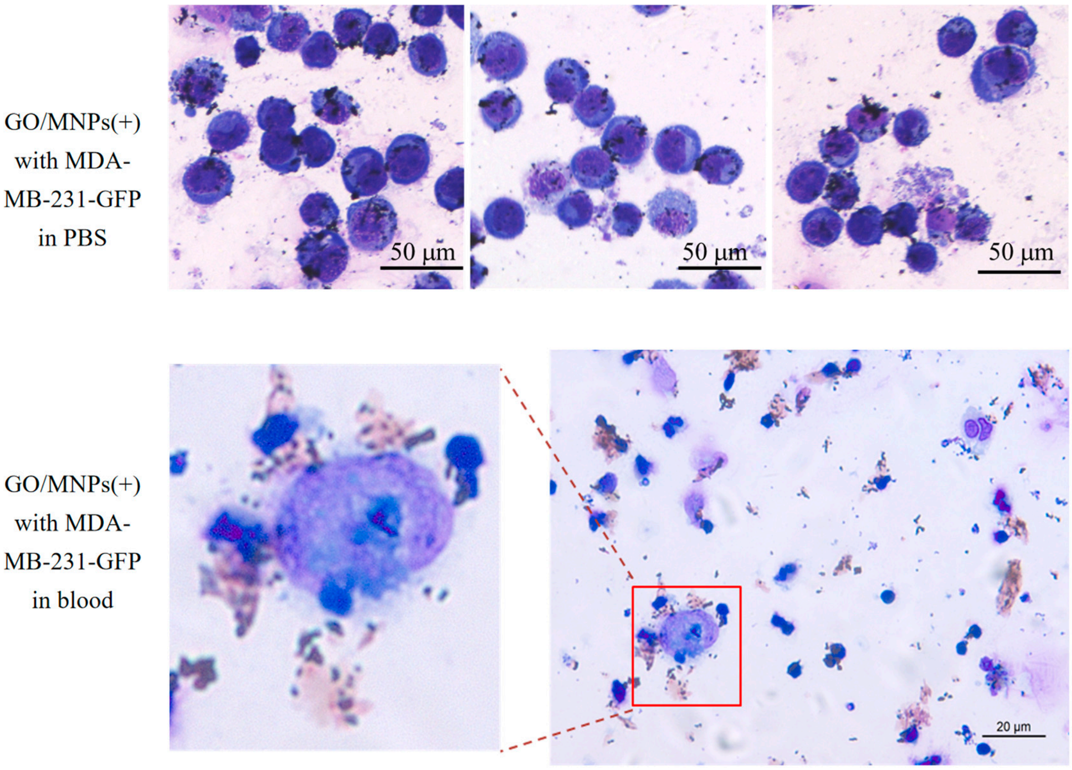

Supplementary Figure S2. The microscopic observation of the specific targeting of GO/MNPs(+) to MDA-MB-231-GFP cells in PBS or blood samples.

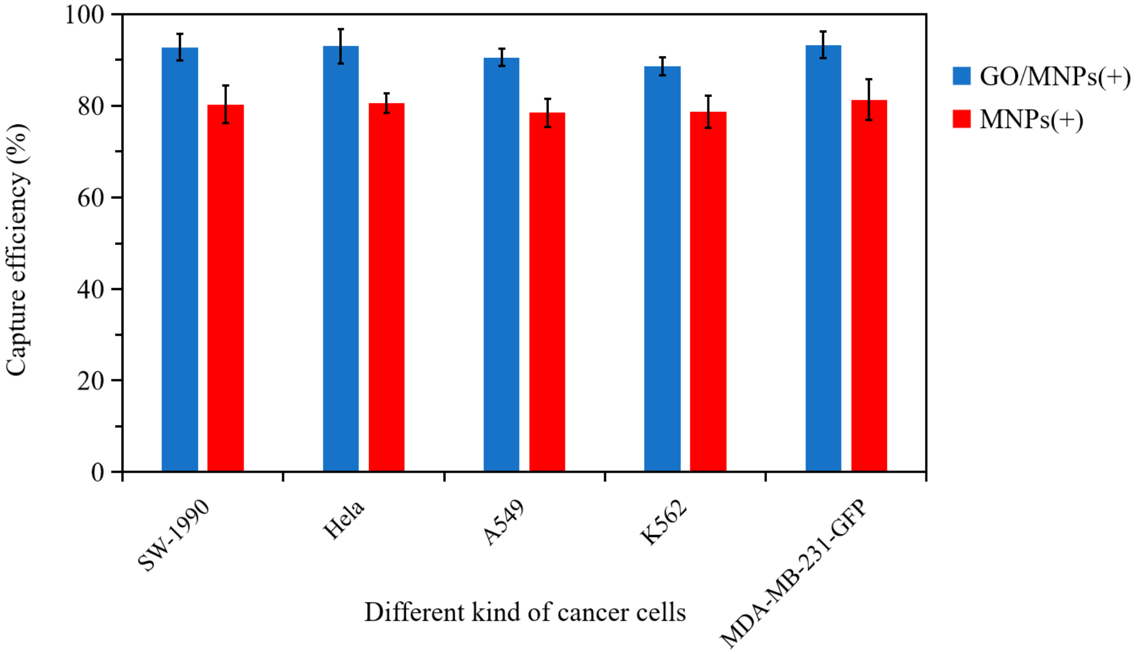

Supplementary Figure S3. Broad-spectrum assay of GO/MNPs(+) and MNPs(+) on five different types of cancer cells.

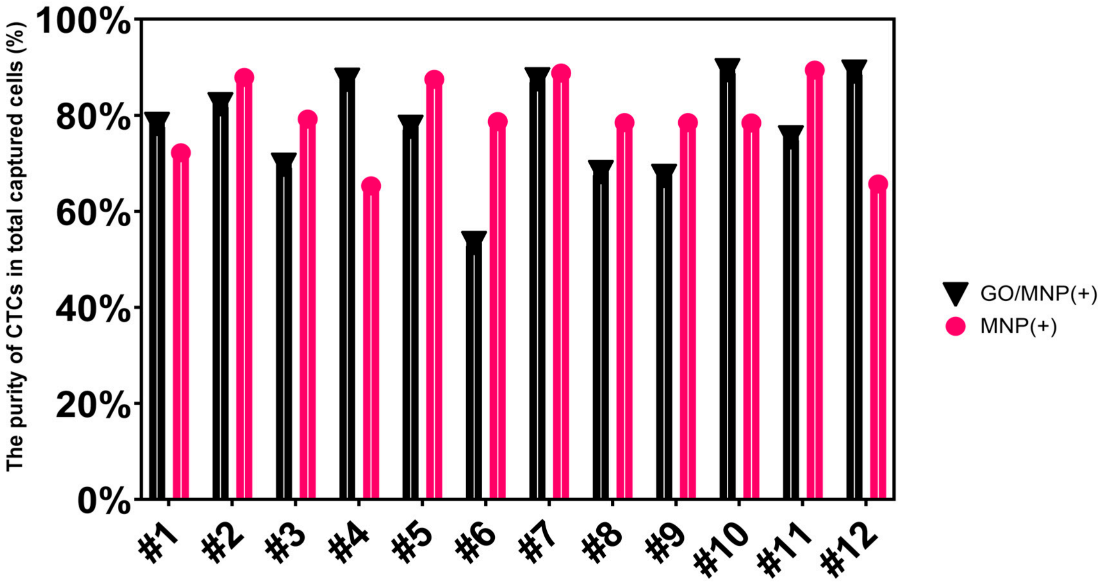

Supplementary Figure S4. The purity of CTCs in total captured cells in clinical samples.

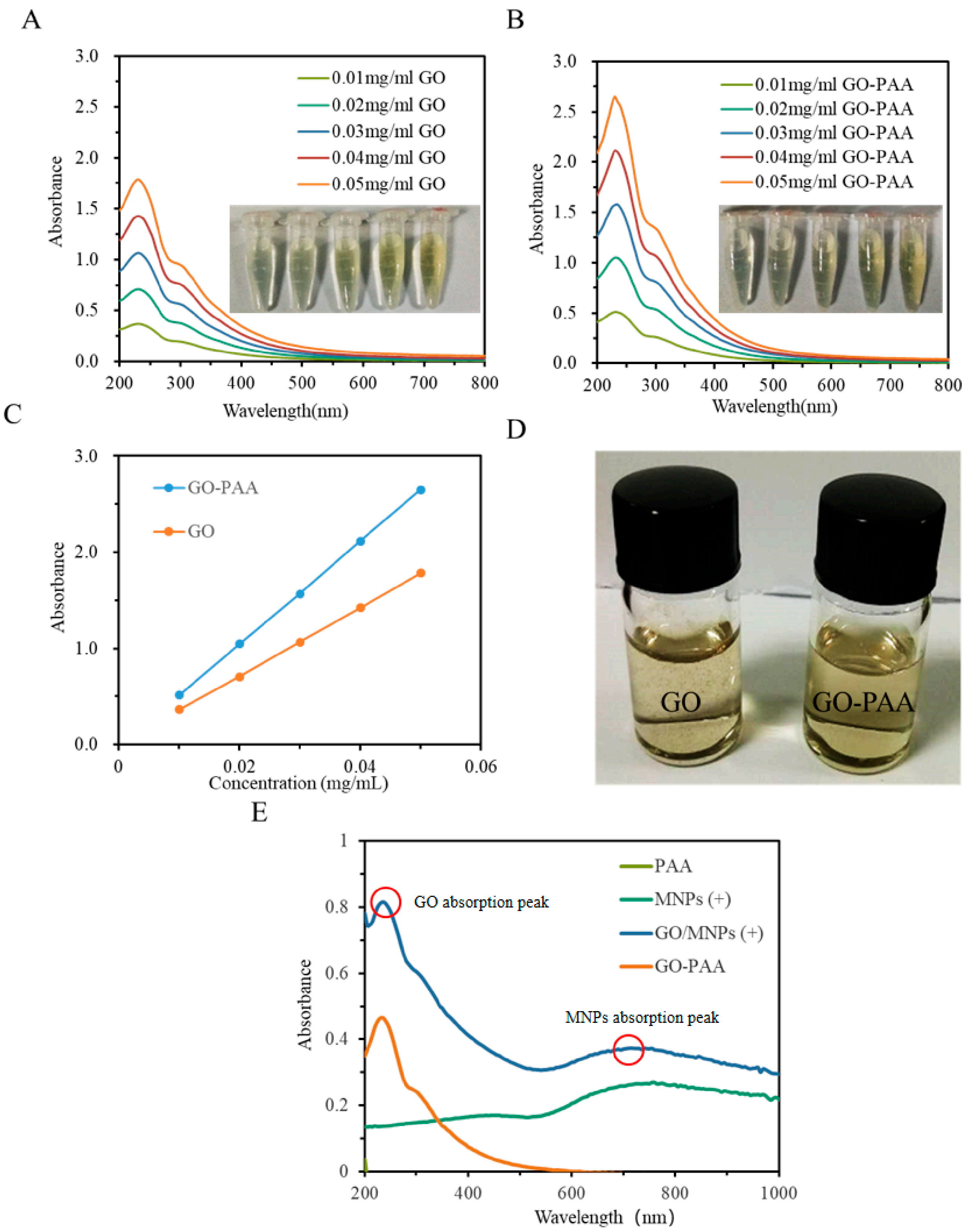

Supplementary Figure S5. UV-Vis absorption spectra of (**A**) GO and (**B**) GO-PAA; (**C**) the curve of the relationship between absorbance and concentration at 230 nm in GO and GO-PAA; (**D**) dispersion of GO and GO-PAA in water for 15 days; (**E**) UV-Vis absorption spectra of GO-PAA, PAA, MNPs(+), GO/MNPs(+).

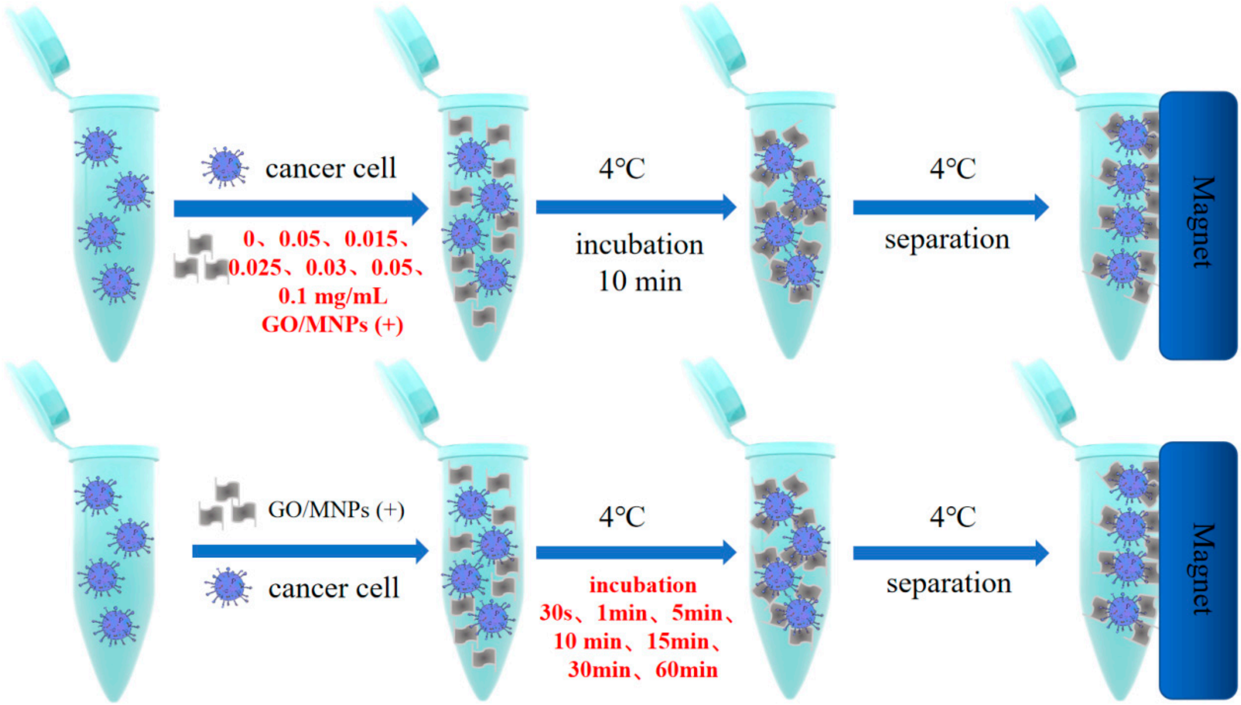

Supplementary Figure S6. Schematic of the optimal conditions for the separation of cancer cells using GO/MNP(+) nanoprobe.

Table S1. Information of clinical samples.

Patient ID	Sex	Clinical Diagnosis
#1	F	Breast cancer
#2	M	Colon cancer
#3	M	Liver cancer
#4	M	Nasopharyngeal cacinoma
#5	M	Melanoma
#6	M	Hepatobiliary cacinoma
#7	F	Nasopharyngeal cacinoma
#8	M	Hepatic metastasis of pancreatic cancer
#9	M	Lung cancer
#10	F	Lung cancer
#11	F	Pancreatic cancer
#12	F	Hepatobiliary cacinoma
